# Comparing the Effect of Spinal Versus General Anesthesia on Postoperative Opioid Use in Minimally Invasive Transforaminal Lumbar Interbody Fusion: A Patient Matched Study

**DOI:** 10.3390/jcm15020781

**Published:** 2026-01-18

**Authors:** Harshvardhan G. Iyer, Jesus E. Sanchez-Garavito, Jorge Rios-Zermeno, Andrew P. Roberts, Juan P. Navarro Garcia de Llano, Loizos Michaelides, Jimena Gonzalez-Salido, Benjamin F. Gruenbaum, Elird Bojaxhi, Oluwaseun O. Akinduro, Ian A. Buchanan, Kingsley O. Abode-Iyamah

**Affiliations:** 1 Department of Neurologic Surgery, Mayo Clinic, Jacksonville, FL 32224, USA; iyer.harshvardhan@mayo.edu (H.G.I.); sanchezgaravito.jesus@mayo.edu (J.E.S.-G.); jriosz@uic.edu (J.R.-Z.); andrewroberts3221@gmail.com (A.P.R.); navarrogarciadellano.juanpablo@mayo.edu (J.P.N.G.d.L.); michaelides.loizos@mayo.edu (L.M.); jimenagonzalezsalido@gmail.com (J.G.-S.); akinduro.oluwaseun@mayo.edu (O.O.A.); buchanan.ian@mayo.edu (I.A.B.); 2 Department of Anesthesiology, Mayo Clinic, Jacksonville, FL 32224, USA; gruenbaum.benjamin@mayo.edu (B.F.G.); bojaxhi.elird@mayo.edu (E.B.)

**Keywords:** awake spine surgery, general anesthesia, minimally invasive, opioids postoperative pain, spinal anesthesia, transforaminal lumbar interbody fusion

## Abstract

**Background/Objectives**: Postoperative opioid exposure after lumbar fusion remains a key clinical concern. Understanding which perioperative factors are associated with lower postoperative opioid use may help optimize recovery after minimally invasive (MIS) transforaminal lumbar interbody fusion (TLIF). This study aimed to determine if patients undergoing MIS-TLIF under spinal anesthesia (SA) showed lower postoperative opioid use compared to those undergoing MIS-TLIF under general anesthesia (GA). **Methods**: We retrospectively studied all adult patients (>18 years) undergoing 1- and contiguous 2-level MIS-TLIFs performed by a single surgeon. Patients undergoing the procedure under GA were compared to those undergoing the procedure under SA. Postoperative oral opioid use, up to 3 months post discharge, was collected. A 1:1 propensity score matching (PSM) protocol was implemented. Each outcome variable was initially assessed using univariate regression. Predictor variables with a *p*-value < 0.2 were included in the multivariate regression model. This was a retrospective, non-randomized study, and residual confounding cannot be excluded despite PSM. **Results**: The matched groups (*n* = 50 in each group) did not differ significantly depending on demographics or levels fused. Before regression, mean number of postoperative opioid prescriptions (*p* = 0.03), mean total operating room (OR) time in minutes (*p* < 0.01), and median length of stay (LOS) in days (*p* = 0.03) were significantly different. Multivariate regression showed that the GA group received 216.5 more total morphine milligram equivalents than the SA group (95% CI = 0.7–432.2, *p* = 0.049). The days of opioid use were higher in the GA group by 3.8 days (95% CI = 0.5 to 7.1, *p* = 0.025). On multivariate regression, LOS in hours was greater in the GA group by 14.1 h (*p* = 0.042). **Conclusions**: SA is an effective anesthetic modality for spinal surgery with the advantages of reduced postoperative opioid use, reduced OR time, and shorter LOS compared to GA.

## 1. Introduction

Transforaminal lumbar interbody fusion (TLIF) is a widely used surgical technique for the treatment of spinal stenosis, grade I and II spondylolisthesis, spinal instability, and degenerative disc disease [1]. As the population ages and the burden of degenerative lumbar spine disease rises, the number of lumbar fusions performed in the United States has increased substantially. Between 2004 and 2015, elective lumbar fusions increased by 62%, reflecting this trend [2]. While TLIFs and other lumbar fusion procedures can improve pain, neurological function, and quality of life, their increased utilization has raised concerns regarding healthcare resource use, perioperative complication rates, and long-term outcomes [2,3,4,5].

A major concern associated with the growing number of lumbar fusions is persistent postoperative pain and chronic opioid dependence. Postoperative pain after lumbar fusions is notoriously difficult to manage, and opioids remain the primary analgesics prescribed at the time of discharge [6]. Although preoperative opioid use is one of the strongest predictors of prolonged postoperative opioid use, large national database and retrospective cohort studies show that 15–18% of opioid naive patients undergoing lumbar fusions continue to fill opioid prescriptions one year after surgery [7,8,9,10]. Consequently, perioperative strategies to minimize opioid requirements while maintaining effective analgesia and recovery have become increasingly important and include multimodal analgesia and enhanced recovery after surgery (ERAS) pathways [11,12,13].

As part of these efforts to optimize perioperative care, lumbar spine surgery has increasingly shifted towards tissue sparing minimally invasive (MIS) techniques since the 1990s [14]. MIS-TLIFs offer advantages over open TLIFs, including reduced soft tissue damage, less blood loss, shorter hospital stay, and lower perioperative complication rates [15]. Building on these advantages, ERAS protocols have been incorporated into MIS-TLIFs, where standardized multimodal analgesia, early mobilization, and streamlined perioperative care have been associated with shorter hospital stays and lower inpatient opioid use, and may facilitate same-day or next-day discharge in carefully selected patients [16,17,18,19].

Along with advances in surgical technique and perioperative protocols, another novel approach that has gained interest is the use of spinal anesthesia (SA), instead of general anesthesia (GA), for MIS-TLIFs. Early experiences suggest that MIS-TLIF under SA is feasible and may be associated with better perioperative and postoperative outcomes compared to GA [20,21]. Studies comparing SA versus GA for MIS-TLIFs report shorter procedure times, earlier ambulation, decreased postoperative fatigue, and lower early postoperative pain scores with SA [20,21,22,23,24,25]. Several reports also describe lower immediate postoperative opioid requirements and, in some cases, reduced opioid consumption during early recovery with SA compared to GA [20,22,25]. In addition, SA has been associated with more stable intraoperative hemodynamics, lower rates of postoperative nausea and vomiting, and shorter length of stay (LOS) [22,23,24,25]. These benefits appear particularly pronounced in patients undergoing 1- or contiguous 2-level MIS-TLIFs where SA has also demonstrated lower complication rates and higher patient satisfaction [20,21,22,23,24]. Yet, GA remains the predominant anesthetic modality for MIS-TLIF, and concerns persist regarding patient movement during surgery, workflows, and team familiarity with surgery under SA [26].

Patients undergoing MIS-TLIF under SA may experience better pain control than those undergoing the procedure under GA, which may translate to lower opioid consumption [27]. However, most existing studies evaluate opioid requirements only during the immediate postoperative period or index hospitalization, leaving outpatient prescribing patterns and cumulative opioid exposure after discharge poorly characterized [20,22,27]. To address this gap, the present study evaluates differences in postoperative opioid consumption between patients undergoing 1-level and contiguous 2-level MIS-TLIFs under SA versus GA. We compare cumulative opioid use up to 3 months postoperatively, as well as outpatient prescribing patterns, between the cohorts. Our goal is to determine whether anesthetic modality is associated with reduced opioid exposure after MIS-TLIF and to inform opioid sparing strategies and future adoption of awake MIS-TLIF in appropriately selected patients.

## 2. Materials and Methods

After Institutional Review Board approval (IRB# 18-003951), we retrospectively reviewed the 1- and contiguous 2-level MIS-TLIFs performed by the same senior spinal surgeon at a tertiary care institution between July 2019 and December 2023, forming a single surgeon retrospective cohort that minimized variability in operative technique and perioperative decision making, while reflecting real-world clinical practice at a high-volume center. We included adult patients (>18 years old) who underwent the procedure for degenerative disc disease, spinal stenosis, and grade I or II degenerative spondylolisthesis, representing the most common degenerative lumbar pathologies for which MIS-TLIF is typically indicated and in which postoperative pain control and functional recovery are critical determinants of outcome. All patients set to undergo the procedure without a history of seizure, intracranial hypertension, anxiety, sleep apnea, compromised airway, previous CSF leak, body mass index (BMI) ≥ 35 kg/m^2^, coagulopathy, infection at the site of needling, and myelographic demonstration of arachnoiditis were offered the choice to undergo surgery under GA or SA, in accordance with our institutional awake spine surgery protocol and previously published criteria for patient selection [25,28,29]. Patients with the above-mentioned contraindications were not excluded, but were offered surgery only under GA, ensuring that clinical eligibility for SA did not restrict access to surgical care while still maintaining patient safety. After counseling by the neurosurgeon and anesthesiologist, and through a shared decision-making discussion that reviewed the potential risks and benefits of GA and SA, patients decided their own anesthetic modality based on their preference. All patients consented prior to surgery, including giving consent for the operative procedure itself and for the use of deidentified clinical data for research and quality improvement purposes in accordance with institutional and IRB guidelines.

**Figure 1:**
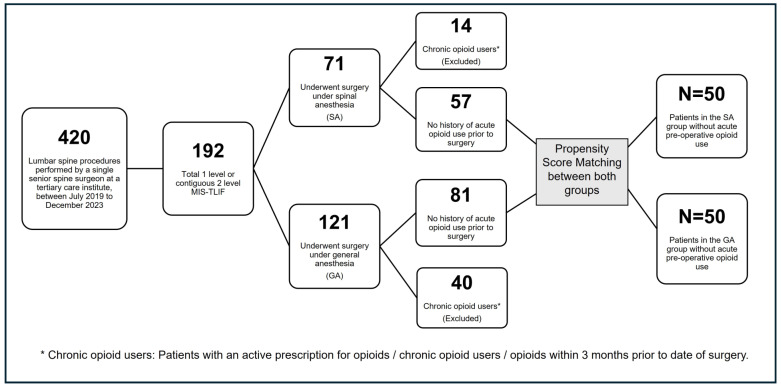
Flow chart outlining the subject selection of 138 adult patients without any history of acute opioid use prior to surgery followed by propensity score matching—100 total matched patients—[50 = spinal anesthesia (SA), 50 = general anesthesia (GA)]. MIS—minimally invasive, TLIF—transforaminal lumbar interbody fusion, SA—spinal anesthesia, GA—general anesthesia.

Among the 192 patients undergoing 1- or contiguous 2-level MIS-TLIF, 121 received GA and 71 received SA, reflecting the gradual but steady adoption of SA for MIS-TLIF within our practice over the study period. Patients with active opioid prescriptions, chronic opioid use, and opioids prescribed in the 3 months prior to surgery were excluded from both the groups, leaving 57 patients in the SA group and 81 in the GA group (Figure 1). This step was taken to ensure that postoperative opioid exposure predominantly reflected new prescriptions given after the MIS-TLIF procedure, rather than continuation of preexisting opioid regimens, and also to avoid the confounding effect of chronic preoperative opioid use, which is one of the strongest predictors of prolonged postoperative opioid use [7,8,9,10]. Oral opioid prescriptions given to the patients at discharge and up to 3 months postoperatively were collected from the electronic medical records (EMRs), including medication name, dose, frequency, quantity dispensed, and intended duration of use, thereby allowing conversion to morphine milligram equivalents (MMEs) and calculation of cumulative exposure measures over time. Inpatient intravenous and oral opioid medications were not included in the study, as the team involved in postoperative care followed the enhanced recovery after surgery protocol, focusing on the use of multimodal analgesic regimens to improve pain control and reduce immediate postoperative opioid consumption [11], and our primary interest was in characterizing outpatient opioid prescribing patterns during the transition from hospital to home rather than short-term inpatient analgesic practices.

### 2.1. Total Morphine Milligram Equivalent (MME) Variable

**Figure 2:**
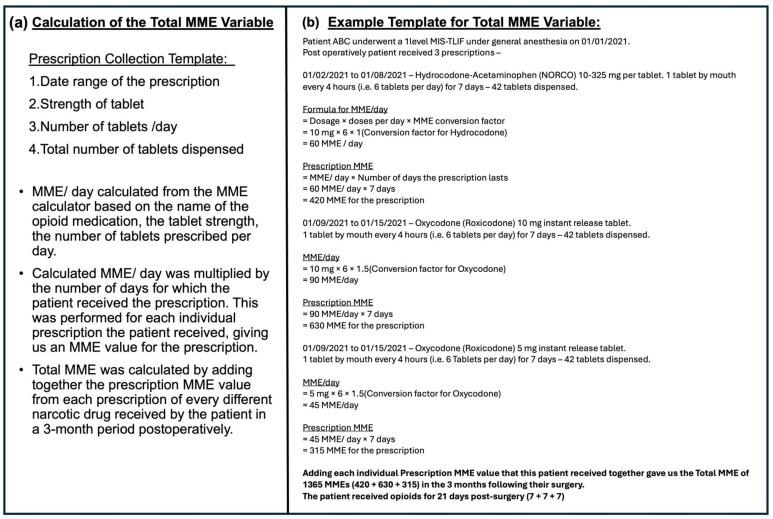
(**a**) Calculation of the total MME variable; (**b**) example template total MME variable. MME—morphine milligram equivalent.

For each prescription, MME/day was calculated using the Centers for Disease Control and Prevention (CDC) Guidelines [30]. The MME/day calculation is an effective way to monitor the daily opioid load, as it standardizes different opioid medications to a common MME scale and allows comparison across patients and time points. However, a major limitation of this metric is that it informs us of only a single day’s opioid usage. It does not inform us about the entire duration of the prescription, nor does it consider the refills and their respective durations, all of which are critical when trying to understand a patient’s overall exposure to opioids after surgery. Hence, we calculated the cumulative opioid use (“Total MME” variable); by summing up the MMEs from every outpatient oral opioid prescription filled within the 3-month postoperative period by the patients (Figure 2). This measure incorporates both the dose and the duration of all the prescriptions, providing a more comprehensive estimate of total postoperative opioid exposure than MME/day alone.

### 2.2. Statistical Analysis

The unpaired *t*-test and the Mann–Whitney U-test were used to compare parametric and non-parametric continuous variables, respectively, when evaluating differences between the GA and SA groups. For categorical outcomes, the chi-square or the Fisher’s exact test were used to compare proportions across groups. To address potential confounders while adjusting for comparison of outcomes between the groups, we applied a 1:1 propensity score matching (PSM) protocol using the K-nearest neighbor method with a caliper width of 0.2. This approach was used to generate matched GA and SA cohorts with more comparable baseline characteristics, thereby reducing imbalances between the groups. Preoperative patient characteristics included in the match were age, sex, race, BMI, and the number of levels fused. Patients were not excluded from the match based on their comorbidities or their symptoms at presentation, allowing these factors to be explored later as independent predictors in regression analyses rather than as matching constraints.

Univariate comparisons of preoperative variables and postoperative outcomes were conducted between the groups, using the same tests mentioned above. To further control for residual confounding, logistic regression was used for binary outcomes, and linear regression was used for continuous outcomes. In the regression analysis, each predictor variable was initially assessed using univariate regression. Predictor variables with a *p*-value of less than 0.2 were included in the multivariate regression model. The final multivariate regression models included variables that remained statistically significant after adjustment. Odds ratios (ORs) with 95% confidence intervals (CIs) were calculated for binary outcomes, while coefficients were determined for continuous outcomes to quantify the association between predictor variables and outcomes. All statistical tests were two-tailed, and a *p*-value of less than 0.05 was considered statistically significant. The statistical analyses were conducted using Python (Python Software Foundation, version 3.11.3) with the SciPy and Stats Model libraries.

## 3. Results

The total cohort included 138 patients without preexisting opioid use. Of these, 81 patients were in the GA group and 57 were in the SA group.

### 3.1. Spinal Anesthesia (SA) Versus General Anesthesia (GA) Cohorts Before PSM

Prior to PSM, there were no significant differences in baseline demographics, comorbidities, and the preoperative symptoms between the SA and GA groups (Table 1). Before matching, the number of contiguous 2-level MIS-TLIFs was higher in the GA group (*p* < 0.01), whereas after matching, the distribution of the 1-level versus 2-level procedures was no longer significantly different between groups. The OR duration was shorter in the SA group, with a mean time of 162.5 min compared to 199.6 min in the GA group (*p* < 0.01). Mean procedure times, calculated from skin incision to closure, were also shorter for SA (121.8 min) than for GA (144.3 min, *p* < 0.01). The mean LOS in the SA group (0.81 days) was significantly shorter in comparison to the GA group (1.88 days, *p* < 0.01).

With respect to outpatient oral opioid use, the median total MME (*p* = 0.13) and median total days of opioid use (*p* = 0.08) were lower in the SA group, although these differences did not reach statistical significance.

### 3.2. Comparing SA Versus GA After PSM

After 1:1 PSM, each group included 50 patients. Besides a higher number of patients with hypertension in the GA group, comorbidities, demographics, and preoperative symptoms were comparable between the groups (Table 2). Importantly, the distribution of 1-level versus contiguous 2-level MIS-TLIFs, which was significantly different before matching, was no longer significantly different between groups after PSM (Table 2).

**Figure 3:**
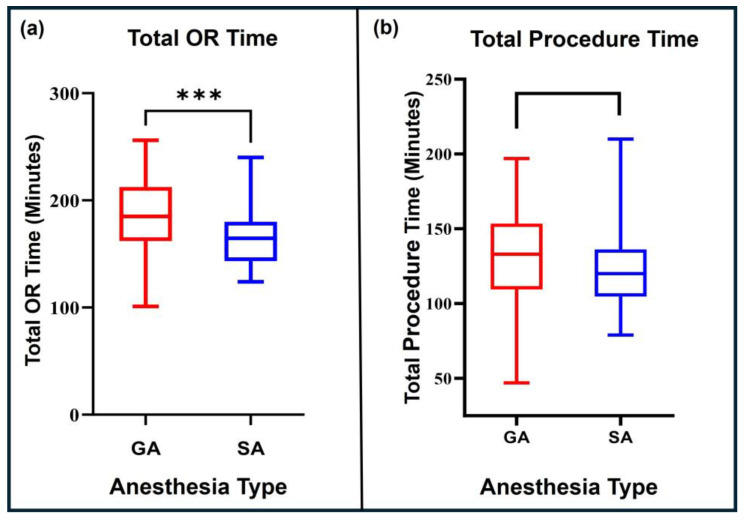
(**a**) Total OR time in minutes compared between the matched GA and SA groups before multivariate regression analyses. (**b**) Total procedure time in minutes compared between the matched GA and SA groups before multivariate regression analyses. *** *p* < 0.01. OR—operating room, GA—general anesthesia, SA—spinal anesthesia.

Mean total OR time remained significantly shorter in the SA group (162.7 min) compared to the GA group (186.7 min, *p* < 0.01) (Figure 3a). In contrast, median surgical procedure time, calculated from skin incision to closure, was 116.5 min in the SA group and 133 min in the GA group, with no significant difference between groups after PSM (*p* = 0.06) (Figure 3b).

**Figure 4:**
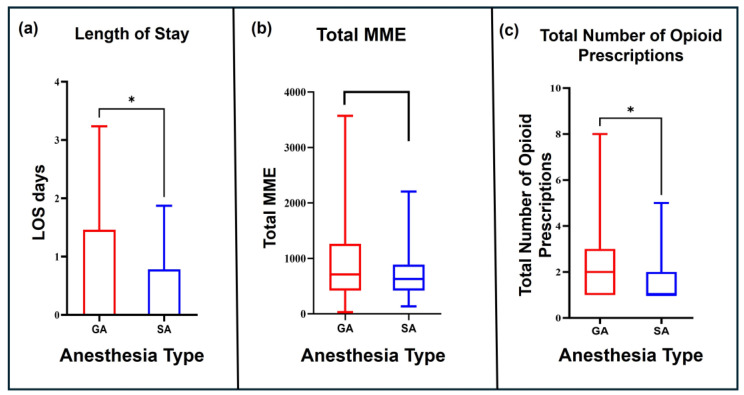
(**a**) Length of stay in days compared between the matched GA and SA groups before multivariate regression analyses. (**b**) Total MMEs consumed in a 3-month postoperative period compared between the matched GA and SA groups before multivariate regression analyses. (**c**) Total number of opioid prescriptions prescribed in a 3-month postoperative period compared between the matched GA and SA groups before multivariate regression analyses; * *p* < 0.05. MME—morphine milligram equivalent, GA—general anesthesia, SA—spinal anesthesia, LOS—length of stay.

The median LOS in days was lower in the SA group (0 days) compared to the GA group (1 day) (*p* = 0.03) (Figure 4a). The mean total MMEs were lower in the SA group (679.5 MMEs) compared to the GA group (885.4 MMEs), although this difference did not reach statistical significance (*p* = 0.07) (Figure 4b). The mean number of opioid prescriptions needed within 3 months post procedure was significantly lower in the SA group (1.66 prescriptions) versus the GA group (2.24 prescriptions, *p* = 0.03) (Figure 4c and Table 3).

### 3.3. Regression Analysis

#### 3.3.1. Total MME

On multivariate linear regression, the GA group received 216.5 more total MMEs than the SA group (95% CI = 0.7–432.2, *p* = 0.049). Total MMEs in patients undergoing contiguous 2-level MIS-TLIFs in both the groups was greater by 418.3 MMEs compared to 1-level MIS-TLIFs (95% CI = 123.96 to 712.66, *p* = 0.006). A higher BMI was also associated with higher total MMEs, with each unit increase in BMI corresponding to an additional 27.6 total MMEs (95% CI–4.7 to 50.5, *p* = 0.019) in both groups (Table 4).

#### 3.3.2. Days of Opioid Use

In the multivariate model, GA patients required an additional 3.8 days of opioid use compared to the SA patients (95% CI = 0.5 to 7.1, *p* = 0.025). The contiguous 2-level procedures in both groups were associated with 5.9 more days of opioid use compared to 1-level procedures (95% CI = 1.4 to 10.4, *p* = 0.011). Every unit rise in BMI was associated with 0.5 additional days of opioid use (95% CI: 0.2 to 0.9, *p* = 0.004).

#### 3.3.3. Number of Opioid Prescriptions

MIS-TLIFs performed under SA were associated with 0.6 fewer opioid prescriptions as compared to MIS-TLIFs under GA (95% CI = 0.1 to 1.1, *p* = 0.02). Contiguous 2-level procedures were associated with 0.9 more prescriptions as compared to 1-level procedures in both the groups (95% CI = 0.4 to 1.5, *p* = 0.015). A higher BMI was associated with 0.07 additional opioid prescriptions in both groups (95% CI = 0.02 to 0.12, *p* = 0.012).

#### 3.3.4. Length of Stay (LOS) in Hours

The patients in the SA group had a 14.1 h shorter LOS as compared to patients in the GA group (95% CI: 0.6 to 27.7, *p* = 0.042). Contiguous 2-level fusions in both groups were associated with 7.7 additional hours of LOS in comparison to 1-level surgeries (95% CI = 1.4 to 13.2, *p* = 0.009). Patients with a history of CAD were associated with 14.9 more hours of LOS in both groups (95% CI: 2.4 to 27.4, *p* = 0.019).

## 4. Discussion

Growing evidence suggests that SA provides advantages over GA in terms of postoperative pain control and recovery in the immediate postoperative period and at long-term follow-up in patients undergoing MIS-TLIFs [20,22,25,31,32]. Our current study adds to this body of work by evaluating whether these benefits translate into reduced postoperative opioid use following 1-level and contiguous 2-level MIS-TLIFs. To our knowledge, this is one of the first matched cohort studies to examine opioid use up to 3 months postoperatively in this context. By focusing exclusively on patients without preexisting chronic opioid use and by examining multiple variables associated with outpatient opioid prescribing like total MME, days of postoperative opioid use, and number of opioid prescriptions, this study provides a more granular view of how anesthetic choice may shape early recovery after MIS-TLIF. In the context of an ongoing opioid crisis, these data could position SA not only as an anesthetic alternative, but also as a potentially impactful perioperative strategy within modern spine surgery [33].

### 4.1. Opioid Use and Pain Management

In our matched cohort, patients undergoing MIS-TLIFs under SA required significantly fewer opioids within the 3-month postoperative period, as reflected by the lower total MME, shorter duration of opioid use, and fewer opioid prescriptions. Because the SA and GA groups were matched on key baseline factors, and patients with preoperative chronic opioid use were excluded, the differences in postoperative opioid use between the groups are less likely to be explained only by obvious baseline imbalances. In addition, all procedures were performed by a single senior surgeon, and the incision to closure times did not differ significantly between the groups, suggesting minimal variability in the operative technique. Taken together, these findings provide supportive evidence suggesting that the choice of anesthesia may be an important contributing factor within an overall opioid sparing perioperative strategy to help reduce postoperative opioid use in patients undergoing 1-level or contiguous 2-level MIS-TLIFs. These findings are consistent with prior studies showing reduced immediate postoperative pain and analgesic requirements in patients undergoing lumbar surgeries under SA compared to GA [20,22,25,27].

A previous study demonstrated lower mean maximum numeric rating scale (NRS) pain scores during the first 3 h in the post-anesthesia care unit (PACU) among SA patients compared to GA, although it did not show a significant difference in the total MMEs administered during the immediate inpatient postoperative period. In their cohort of patients, similar narcotic requirements despite lower NRS pain scores in the SA group, as compared to the GA group, were interpreted as evidence of better pain control with the same opioid dosage across groups. The authors also acknowledge that the small sample size and potential differences in pain tolerance between groups could have influenced these findings [20]. Another prospective cohort study found sustained improvements in lower back, leg pain, and Oswestry Disability Index scores up to 12 months postoperatively in patients undergoing MIS-TLIFs under SA as compared to GA [32]. Our findings support these trends and further demonstrate that the benefits of SA may extend beyond the early postoperative period.

A smaller matched series of 20 patients also found opioid use to be lower with SA, compared to GA, for 1-level and 2-level MIS-TLIFs; however, the use of two different MIS-TLIF techniques and a 6 h postoperative observation window may limit the generalizability of those findings [27]. Compared to this earlier work, our study extends the postoperative opioid use observation window to 3 months after surgery, which is more closely linked to the risk of transition from acute to persistent opioid use [33,34].

Evidence from orthopedic procedures, particularly hip and knee arthroplasties, has shown that SA is associated with reduced intraoperative and postoperative narcotic use in the PACU [31,35,36]. For example, one matched cohort study of patients undergoing outpatient total hip and knee arthroplasty at an ambulatory surgery center showed that patients receiving SA consumed fewer MMEs in the PACU than those receiving GA [35]. Another study reported that patients undergoing joint arthroplasty under GA needed higher doses of both opioids and non-opioid adjuncts and still reported more pain compared to SA [36]. These consistent findings across surgical specialties support a broader application of SA in select spine procedures.

### 4.2. Procedure Time and Discharge

We found no significant difference in TLIF procedure times between the SA and GA groups, suggesting that the surgical workflow remained consistent regardless of the anesthetic modality. However, the mean total OR time, which includes the time taken for anesthesia induction along with the surgery time, was significantly longer in the GA group. This highlights the potential for SA to reduce overall procedural time through more efficient perioperative management.

Patients in the SA group had a shorter median hospital stay in days. The median LOS was 0 days for SA patients compared to 1 day for the GA patients, reflecting that many SA patients in our cohort were discharged on the same day as the procedure. After PSM, the univariate comparison of LOS in hours did not reach statistical significance; however, LOS in hours was significantly shorter for the SA group on multivariable regression. This is consistent with previous studies in orthopedics, and in spine surgery that have reported reduced LOS in patients undergoing surgery under SA as compared to GA [22,27,37,38]. Within the broader framework of value-based care and ERAS pathways, shorter OR times and LOS are clinically meaningful because they can improve the efficiency of OR use, allow earlier mobilization, and support a faster return to baseline activity. For 1- and 2-level MIS-TLIFs, where many patients may be candidates for short-stay or ambulatory care, combining SA with MIS techniques and ERAS-based postoperative management may offer a practical route to expanding outpatient lumbar fusion programs without compromising patient safety. Despite these well-documented benefits, GA is still the primary anesthetic modality of choice for these procedures [26].

### 4.3. Body Mass Index (BMI) and Postoperative Opioid Use

In our multivariable regression analyses of the matched cohort, higher BMI was associated with greater oral opioid exposure during the 3-month postoperative period, including higher total MMEs, longer duration of opioid use, and a greater number of opioid prescriptions. This association was observed across the entire matched cohort and was not specific to either anesthetic group, suggesting that patient level factors also play an important role in postoperative opioid exposure.

These findings are supported by prior studies of lumbar spine surgery that have shown higher BMI to be associated with greater postoperative opioid prescribing and opioid consumption after lumbar spine surgery [39,40]. Evidence from MIS-TLIF cohorts also suggests that morbid obesity is associated with greater postoperative analgesic requirements and longer length of stay, supporting a higher recovery burden in these patients [41]. Taken together, these data emphasize that postoperative opioid exposure is multifactorial, and BMI represents an important patient level contributor that should be considered alongside anesthetic modality when interpreting opioid-related outcomes after MIS-TLIF.

### 4.4. Clinical Advances and Future Directions

Taken together, our findings suggest that SA for 1- and 2-level MIS-TLIF may represent a meaningful clinical advance in the management of degenerative lumbar spine pathologies. Within a standardized MIS technique and ERAS-based perioperative pathway, SA was associated with lower postoperative opioid use, fewer opioid prescriptions, shorter LOS, and no increase in procedure times, compared with GA. In appropriately selected patients, this combined approach may help improve postoperative pain control and overall recovery.

For practicing spine surgeons and anesthesiologists, these results suggest that anesthetic modality is not just a background variable, but a modifiable part of the care pathway that can be integrated into opioid sparing strategies. In patients undergoing 1- or contiguous 2-level MIS-TLIFs, incorporating SA into existing ERAS pathways may help align everyday practice with institutional goals related to opioid stewardship and the development of short-stay or outpatient spine surgery programs.

Despite these potential advantages, SA for MIS-TLIFs remains underutilized, and GA continues to be the primary anesthetic modality used in most centers. A survey-based study reported that many spine surgeons are reluctant to adopt SA due to uncertainty about its advantages over GA. Notably, the survey found that a randomized patient-centered trial could meaningfully influence surgeons’ attitude and increase the adoption of SA for spine procedures [26]. This gap between evidence and adoption highlights that technical feasibility and outcome data alone are not sufficient to change practice. Successful implementation of SA for MIS-TLIF requires coordinated engagement among surgeons, anesthesiologists, nursing staff, and institutional leadership, as well as structured pathways for patient selection, education, and intraoperative communication.

Future studies should evaluate longer-term opioid use beyond 3 months and incorporate patient reported outcomes such as pain, function, fatigue, and satisfaction. Additionally, patient factors, such as BMI, that may affect postoperative opioid use after MIS-TLIF should be evaluated across anesthetic pathways in future studies. Work focused on the implementation of SA in MIS-TLIF is also needed to assess how it performs in different practice environments, including community hospitals and ambulatory surgery centers, and to identify practical barriers and facilitators to its wider adoption. Finally, examining the economic impact of SA-based protocols, including potential reductions in OR time, LOS, and opioid-related complications, will help define their role in value-based spine care and support the development of scalable outpatient lumbar spine surgery programs.

### 4.5. Limitations

We acknowledge the inherent limitations of our study. Given its retrospective, non-randomized design and a relatively small sample size, generalizability is limited and the analysis may be underpowered to detect small differences between the two groups. Selection bias is possible, as patients who preferred GA or those that did not qualify for SA due to contraindications underwent surgery under GA. Residual confounding from unmeasured variables cannot be excluded despite PSM. Although the single-surgeon, single-institution design reduces variability in operative technique and perioperative workflow, it may limit external validity, as anesthesia selection practices and perioperative care pathways can differ across surgeons and institutions. Successful implementation of SA for MIS-TLIF also depends on institutional experience and coordinated anesthesiology and surgery workflows, and occasional conversion to GA may be necessary. Importantly, we excluded patients with chronic preoperative opioid use, as preexisting opioid dependence is the strongest and most consistent predictor of postoperative opioid consumption [42,43]. A limitation of opioid data collection was that we tracked the filled prescriptions and not the pills actually taken, so true opioid use might differ. In addition, patient-reported outcome measures were not collected, which limits the assessment of pain and functional recovery alongside opioid use. Nevertheless, we employed PSM to reduce confounding and ensure baseline comparability between the SA and GA groups, strengthening the validity of our findings.

## 5. Conclusions

Opioid misuse and overdose remain critical public health issues in the United States, with opioid naive patients undergoing TLIFs at increased risk for developing dependence [44]. Our matched cohort analysis demonstrates that SA is a viable and effective anesthetic modality for MIS-TLIFs, offering important advantages over GA, including reduced postoperative opioid use, shorter OR times, and shorter hospital LOS. These findings support the growing body of literature advocating for the expanded use of SA in select spine procedures. The use of SA for MIS-TLIF may represent an important avenue for clinical innovation and value-based care in lumbar spine surgery. Future prospective, randomized trials are needed to validate these results and assess whether SA can meaningfully reduce the rates of opioid dependence and complications in both the short and long term. As pain management continues to evolve, the choice of anesthetic modality should be recognized as a key factor influencing patient recovery and long-term outcomes.

## Figures and Tables

**Table 1:** Preoperative patient characteristics of complete cohort before PSM.

Preoperative Variables	Spinal Anesthesia [SA] (*n* = 57)	General Anesthesia [GA] (*n* = 81)	*p* Value
**Demographics**			
Age (years) (mean)	63.5 ± 10.8	64.3 ± 10.9	0.68 ^††^
BMI (mean)	29.5 ± 4.9	29.8 ± 5.6	0.78 ^†^
**Sex**			0.95 ^
Females	30 (52.6%)	41 (50.6%)	
Males	27 (47.4%)	40 (49.4%)	
**Race**			0.24 ^
Caucasian	45 (78.9%)	71 (87.6%)	
African American	5 (8.8%)	5 (6.2%)	
Hispanic	5 (8.8%)	2 (2.5%)	
Asian	0 (0%)	2 (2.5%)	
Other	2 (3.5%)	1 (1.2%)	
**Number of Levels Fused**			<0.01 ^*
1-level MIS-TLIF	49 (86%)	46 (56.8%)	
2-level MIS-TLIF	8 (14%)	35 (43.2%)	
**Comorbidities**			
Coronary artery disease (CAD)	12 (21.1%)	15 (18.5%)	0.87 ^
Congestive heart failure (CHF)	1 (1.8%)	4 (4.9%)	0.60 ^
Atrial fibrillation	2 (3.5%)	8 (9.8%)	0.27 ^
Stroke	3 (5.3%)	5 (6.2%)	1 ^
Type II diabetes mellitus	12 (21.1%)	12 (14.8%)	0.46 ^
Chronic obstructive pulmonary disease (COPD)	0 (0%)	4 (4.9%)	0.24 ^
Hypertension	24 (42.1%)	46 (56.8%)	0.12 ^
Obesity	29 (50.9%)	37 (45.7%)	0.66 ^
Patients on anticoagulants	2 (3.5%)	7 (8.6%)	0.31 ^
Deep vein thrombosis	2 (3.5%)	0 (0%)	0.32 ^
Sleep apnea	9 (15.8%)	13 (16%)	1 ^
Pulmonary embolism	1 (1.8%)	5 (6.2%)	0.40 ^
**Preoperative Symptoms at Presentation**			
Motor deficit	13 (22.8%)	26 (32.1%)	0.31 ^
Sensory deficit	57 (100%)	81 (100%)	1 ^
Pain	56 (98.2%)	81 (100%)	0.85 ^
**Postoperative Outcomes**			
Mean total OR time in minutes (anesthesia + procedure time)	162.5 ± 26.9	199.6 ± 40.3	<0.01 ^†^*
Mean total TLIF procedure time (minutes)	121.8 ± 28.6	144.3 ± 38.6	<0.01 ^†^*
Mean length of stay (LOS) (days)	0.81 ± 1.1	1.88 ± 2.2	<0.01 ^†^*
Discharge status			0.23 ^
Home	57 (100%)	77 (95%)	
Rehabilitation	0 (0%)	3 (3.7%)	
Nursing facility	0 (0%)	1 (1.3%)	
Opioids required during hospital stay	56 (98.2%)	75 (92.6%)	0.27 ^
Opioids used for >7 days	28 (49.1%)	48 (59.3%)	0.22 ^
Median total MME (range)	630 (135–2940)	720 (30–3570)	0.13 ^†^
Median number of prescriptions (range)	1 (1–5)	2 (1–8)	0.07 ^†^
Median total days of opioid use (range)	7 (3–35)	14 (3–56)	0.08 ^††^
Median average MME/day (range)	60 (25–90)	60 (10–135)	1 ^††^

^†^ *t*-test, ^††^ Mann–Whitney U-test, ^ chi square, * *p* < 0.05 (statistically significant). SA—spinal anesthesia, GA—general anesthesia, MIS—minimally invasive, TLIF—transforaminal lumbar interbody fusion, BMI—body mass index, CAD—coronary artery disease, CHF—congestive heart failure, COPD—chronic obstructive pulmonary disease, OR—operating room, LOS—length of stay, MME—morphine milligram equivalent.

**Table 2:** Comparison between patient characteristics after PSM.

Preoperative Variables Post Matching	Spinal Anesthesia [SA] (*n* = 50)	General Anesthesia [GA] (*n* = 50)	*p* Value
**Demographics**			
Age (mean)	63.5 ± 11.3	62.5 ± 11.9	0.84 ^††^
BMI (mean)	29.4 ± 5.1	28.2 ± 4.9	0.30 ^†^
**Sex**			0.84 ^
Female	26 (52%)	24 (48%)	
Male	24 (48%)	26 (52%)	
Race (Caucasian)	42 (84%)	42 (84%)	0.61 ^
**Number of Levels Fused**			0.78 ^
1-level MIS-TLIF	43 (86%)	41 (82%)	
2-level MIS-TLIF	7 (14%)	9 (18%)	
**Comorbidities**			
CAD	10 (20%)	7 (14%)	0.59 ^
CHF	0 (0%)	2 (4%)	0.47 ^
Atrial fibrillation	1 (2%)	4 (8%)	0.35 ^
Stroke	3 (6%)	2 (4%)	1 ^
Type II diabetes mellitus	10 (20%)	6 (12%)	0.41 ^
COPD	0 (0%)	3 (6%)	0.24 ^
HTN	17 (34%)	32 (64%)	<0.05 ^*
Obesity	26 (52%)	22 (44%)	0.54 ^
Anticoagulants	1 (2%)	5 (10%)	0.20 ^
DVT	2 (4%)	0 (0%)	0.47 ^
Sleep apnea	7 (14%)	6 (12%)	1 ^
Pulmonary embolism	1 (2%)	3 (6%)	0.60 ^
**Preoperative Symptoms at Presentation**			
Motor deficit	13 (26%)	17 (34%)	0.51 ^
Sensory deficit	0 (0%)	0	1 ^
Pain	49 (98%)	50 (100%)	1 ^

^†^ *t*-test, ^††^ Mann–Whitney U-test, ^ chi square, * *p* < 0.05 (statistically significant). SA—spinal anesthesia, GA—general anesthesia, MIS—minimally invasive, TLIF—transforaminal lumbar interbody fusion, BMI—body mass index, CAD—coronary artery disease, CHF—congestive heart failure, COPD—chronic obstructive pulmonary disease, HTN—hypertension, DVT—deep vein thrombosis.

**Table 3:** Comparison between patient outcomes between SA and GA groups after PSM.

Outcome Variable Post Matching:	SA	GA	*p* Value
**Mean Total OR time (min)**	**162.7 ± 27.6**	**186.7 ± 35.8**	<0.01 ^†^*
Median procedure time (min) (range)	116.5 (79–210)	133 (47–197)	0.06 ^††^
Median EBL in cc (range)	30 (1–250)	50 (3–200)	0.17 ^††^
**Median LOS in days (range)**	**0 (0–5)**	**1 (0–7)**	0.03 ^††^*
Median LOS in hours (range)	16.5 (0–120)	24.0 (0–168)	0.07 ^††^
**Discharge status**			0.47 ^
Home	50 (100%)	48 (96%)	
Rehab	0	2 (4%)	
Complications	0 (0%)	1 (2%)	1 ^
Opioids required during hospitalization	50 (100%)	46 (92%)	0.12 ^
PO fatigue	2 (4%)	0 (0%)	0.47 ^
Opioid PACU	42 (84%)	39 (78%)	0.61 ^
Mean total MME	679.5 ± 407.4	885.4 ± 692.1	0.07 ^†^
**Mean total number of opioid prescriptions**	**1.66 ± 0.9**	**2.24 ± 1.6**	0.03 ^†^*
Median days opioid use (range)	7 (3–35)	14 (3–56)	0.15 ^††^
Median average MME/day (range)	60 (25–180)	60 (10–105)	0.15 ^††^
>7 days of opioid	22	29	0.23 ^

^†^ *t*-test, ^††^ Mann–Whitney U-test, ^ chi square, * *p* < 0.05 (statistically significant). SA—spinal anesthesia, GA—general anesthesia, OR—operating room, EBL—estimated blood loss, LOS—length of stay, PO—postoperative, PACU—postoperative anesthesia care unit, MME—morphine milligram equivalents.

**Table 4:** Multivariate regression analysis results for outcomes of interest in the matched cohort.

Outcomes of Interest	Coefficient (95% CI)	*p* Value
**Linear Regression**		
**Total MME**		
SA versus GA	216.5 (0.7–432.2)	0.049 *
2-level versus 1-level TLIF (SA + GA)	418.3 (123.9 to 712.6)	0.006 *
BMI	27.6 (4.7–50.5)	0.019 *
**Total Days of Opioids Use**		
SA versus GA	3.8 (0.5–7.1)	0.025 *
2-level versus 1-level TLIF (SA + GA)	5.9 (1.4–10.4)	0.011 *
BMI	0.5 (0.2–0.9)	0.004 *
**Number of Opioid Prescriptions**		
SA versus GA	0.6 (0.1–1.1)	0.02 *
2-level versus 1-level TLIF (SA + GA)	0.9 (0.4–1.5)	0.015 *
BMI	0.07 (0.02–0.12)	0.012 *
**LOS (Hours)**		
SA versus GA	14.1 (0.6–27.7)	0.042 *
2-level versus 1-level TLIF (SA + GA)	7.7 (1.4–13.2)	0.009 *
CAD	14.9 (2.4–27.4)	0.019 *
**Outcome of Interest**	**OR (95% CI)**	***p*** **value**
**Logistic Regression**		
**>7 Days of Opioid Use**		
BMI	0.15 (0.05–0.25)	0.002 *

* *p* < 0.05 (statistically significant). CI—confidence interval, MME—morphine milligram equivalent, SA—spinal anesthesia, GA—general anesthesia, BMI—body mass index, CAD—coronary artery disease.

## Data Availability

Due to patient privacy and institutional policy, the underlying data from this retrospective study are not publicly available. Deidentified data may be shared upon reasonable request to the corresponding author and with approval from the Institutional Review Board and a data use agreement.

## Abbreviations

The following abbreviations are used in this manuscript:

MIS	Minimally invasive
TLIF	Transforaminal lumbar interbody fusion
SA	Spinal anesthesia
GA	General anesthesia
PSM	Propensity score matching
OR	Operating room
MME	Morphine milligram equivalent
CI	Confidence interval
EMRs	Electronic medical records
LOS	Length of stay
BMI	Body mass index
